# Salinity Gradient Controls Microbial Community Structure and Assembly in Coastal Solar Salterns

**DOI:** 10.3390/genes13020385

**Published:** 2022-02-21

**Authors:** Tianran Song, Qiyun Liang, Zhaozhong Du, Xiaoqun Wang, Guanjun Chen, Zongjun Du, Dashuai Mu

**Affiliations:** 1 SDU-ANU Joint Science College, Shandong University, Weihai 264209, China; 201900700242@mail.sdu.edu.cn; 2 College of Marine Science, Shandong University, Weihai 264209, China; qfsfdxlqy@163.com (Q.L.); duzhaozhong1996@163.com (Z.D.); sduwxq@163.com (X.W.); guanjun@sdu.edu.cn (G.C.)

**Keywords:** multi-pond saltern, salinity gradients, microbial community, assembly, ecological processes

## Abstract

Salinity acts as a critical environmental filter on microbial communities in natural systems, negatively affecting microbial diversity. However, how salinity affects microbial community assembly remains unclear. This study used Wendeng multi-pond saltern as a model to evaluate the prokaryotic community composition and diversity and quantify the relative importance of ecological processes across salinity gradients. The results showed that low-saline salterns (45–80 g/L) exhibited higher bacterial diversity than high-saline salterns (175–265 g/L). The relative abundance of taxa assigned to Halomicrobiaceae, Rhodobacteraceae, Saprospiraceae, and Thiotrichaceae exhibited a hump-shaped dependence on increasing salinity. Salinity and pH were the primary environmental factors that directly or indirectly determined the composition and diversity of prokaryotic communities. Microbial co-occurrence network dynamics were more complex in the sediment than in the water of salterns. An infer Community Assembly Mechanisms by Phylogenetic-bin-based null model analysis (iCAMP) showed that microbial community assembly in sediment and water differed. Our findings provide more information about microbial community structure and the importance of various ecological processes in controlling microbial community diversity and succession along salinity gradients in water and sediment.

## 1. Introduction

Salinity acts as a critical environmental filter on microbial communities in ecosystems [1,2]. In soil environments, many studies indicated that soil salinization could significantly influence microbial communities’ biological structure and functions [3]. Soil salinity limits water availability to plants and microorganisms, thus acting as a stressor. In aquatic environments, salinity was a critical factor in shaping microbial diversity and community structure [4]. At a global scale, salinity has been demonstrated to be one of the essential factors affecting microbial distribution [5].

Some studies showed that salinity is the crucial environmental filter in the assembly of soil microbial communities along with salinity gradients [3,6]. However, whether or how salinity gradients affect microbial communities’ assembly in aquatic and sediment environments remains unknown. Understanding community assembly processes is vital to find the potential factors governing microbial community structure [7,8]. Unraveling the drivers controlling community assembly is a central issue in ecology [8]. It is known that both deterministic (e.g., homogeneous selection and heterogeneous selection) and stochastic processes (e.g., homogenizing dispersal, dispersal limitation, and ‘drift’) affect the assembly of microbial communities. Deterministic processes refer to environment filtering or biotic interactions, while stochastic processes refer to passive dispersal and random demographic changes in mortality [7,8]. By examining deviations from infer Community Assembly Mechanisms by Phylogenetic-bin-based null model analysis (iCAMP), changes in the relative importance of various processes affecting microbial communities can be quantified [9]. Recent studies investigated community assembly processes along with aridity [10] and pH [11] gradients, but little is known about microbial community assembly processes with respect to a salinity gradient.

Multi-pond salterns are semi-artificial coastal systems designed to harvest NaCl from seawater. In this system, seawater is pumped through multi-shallow ponds, in which it is gradually driven to ponds of greater salinities, ranging from that of seawater to sodium chloride saturation and sometimes even beyond [12]. These systems are well known as continuous or semi-continuous systems because each set of ponds maintains a range of salinity for a relatively long time. Many ecological changes happen through this gradient; for example, biodiversity decreases with the increase in salinity [12]. There are many ecological and microbiological studies on the ecology of multi-pond salterns, to the extent that these salterns can be used as model systems for the variations induced by environmental factors [13].

In multi-pond salterns, salinity could play an important role in shaping microbial community composition, and it was found that microbial diversity decreased as salinity increased [14]. However, this pattern is not consistently observed for all microbial communities [15]. It is a long-standing goal and challenge for ecologists to better understand the microbial taxonomic composition and diversity in multi-pond salterns and the mechanisms that shape community structure [16]. In microbial ecology, it has been proposed that “everything is everywhere, but the environment selects,” which suggests that a variation in environmental factors could drive biogeographic patterns of microbial community composition [17]. Microbial diversities or communities are altered by environmental factors such as temperature, salinity, and biological factors [18,19]. In some ecosystems, community composition changes quickly along with the rapid changing of environmental factors. These changes may reflect the rapid growth or dispersal of rare or dormant taxa from a “seed bank” [20,21]. Many studies indicate that dispersal between distant environments is limited [22,23], implying that microbial communities could also be governed by their demographic history. 

The Wendeng solar saltern is a multi-pond saltern that originated from seawater and comprises a set of shallow ponds, where water gradually evaporates, and salts concentrate. Salinity (35 to 300 g/L) and physicochemical factors in these solar salterns vary greatly. They offer the best possible opportunity to rule out the geographical isolation effect, thereby providing a valuable model to investigate the relationship between microbial community composition and various environmental factors in solar salterns. 

The objectives of this study were to (a) compare the microbial community composition and diversity in multi-pond solar salterns using 16S rRNA gene amplicons; (b) evaluate the distribution patterns of the microbial community composition and across entire prokaryotic communities along with salinity and other environmental factors; (c) determine how salinity affects microbial community assembly processes in water and sediments.

## 2. Materials and Methods

### 2.1. Sample Collection

**Figure 1:**
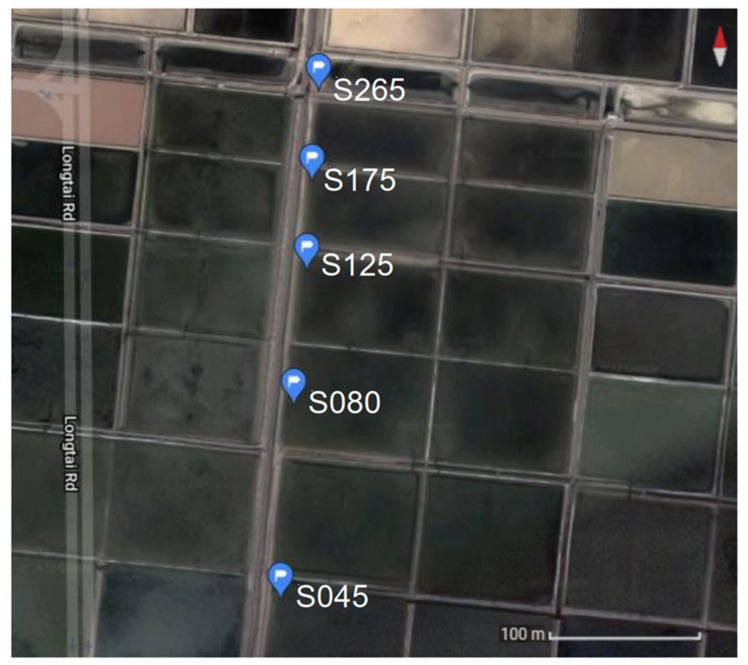
Overview of the Wendeng salterns. The salterns consist of a set of shallow ponds. The salinity of these shallow ponds is about 40, 80, 125, 175, and 265 g/L in sampling sites S045, S080, S125, S175, and S265, respectively.

In May 2019, samples were obtained from Wendeng multi-pond saltern (Weihai, China) and included samples from five ponds with salinity of 45, 80, 125, 175, and 265‰ (S045 36°59’23.6” N 122°02’23.9” E, S080 36°59’27.8” N 122°02’24.3” E, S125 36°59’30.7” N 122°02’24.6” E, S175 36°59’32.7” N 122°02’24.8” E, S265 36°59’34.6” N 122°02’24.9” E). Sediment and water samples were taken from each pond (Figure 1). For every pond, water salinity and pH values were measured in situ [12]. Each pond was sampled in three random locations by collecting sediment and water samples. The water samples from the same pond were pooled and concentrated from 3 L to 500 mL by a hollow fiber membrane module (pore size: 0.22 μm) and then collected in a 500 mL sterile opaque polypropylene bottle; the sediment samples from the same pond were pooled from 500 mL of sterile water. After collection, all samples were instantly sent to the laboratory, kept at 4 °C during the transportation, and stored at −80 °C after treatment. 

### 2.2. Measurement of Physicochemical Factors

The sediment samples were put in a Petri dish and dried it at 105 °C for 6 h. After air-drying, the sediment extract was obtained in the ratio of water to soil of 2.5:1, and the pH of the sediment was measured in the extract. An amount of 5 g of dried sediment samples was put into a 50 mL centrifuge tube, 25 mL of water was added, and the mixture was shaken for 3 min to obtain a 5:1 water–soil extract. Then, the water–soil extract and the water samples from the salterns were filtered by 0.22 μm polyether sulfone membranes. The soluble ion (including Cl^−^, Br^−^, SO_4_^2−^, Na^+^, NH^4+^, K^+^, Mg^2+^, and Ca^2+^) concentration was measured by ICS-1100 (Thermo, Sunnyvale, CA, USA). 

### 2.3. Genomic DNA Extraction and Sequencing

All treated samples were centrifuged to remove particulate matter, and the supernatant fraction was filtered by polyether sulfone membranes to obtain microorganisms with a size greater than 0.22 μm. These membranes were kept at −80 °C before use. Equivalent volumes of samples (with different salinities) were dissolved prior to DNA extraction using a FastDNA Spin Kit for soil (MP Biomedical, France). The prime sets composed of 338F (5′-ACTCCTACGGGAGGCAGCAG-3′) and 806R (5′-GGACTACHVGGGTWTCTAAT-3′) were selected for microbial community structure analysis. Sequencing was carried out on a MiSeq PE300 platform at the Shanghai Majorbio Bio-pharm Technology Co., Ltd. (Shanghai, China). 

### 2.4. Sequence Analysis

The pipeline of vsearch v1.2.11 was employed for quality trimming and removing chimeric sequences [24]. Operational taxonomic units (OTUs) were clustered on the basis of a cut-off value of 97%, and taxonomic annotation was obtained using the SILVA_138_SSU_RefNR99 database [25,26], which was followed by vsearch [24]. The alignment of OTU sequences was done by mafft v7.450 [27], and the phylogenetic tree was constructed by using FastTree [28]. 

The α-diversity (Shannon–Wiener and Simpson diversity indexes) and Venn diagrams (showing shared and unique OTUs) were calculated by the R package “microeco” [29]. Tukey HSD test with ANOVA to analyze α diversities of sediment and water samples was employed. Meanwhile, for distinguishing the general distribution patterns of the prokaryotic community composition in the sediment and water samples of the saltern, non-metric multidimensional scaling (NMDS) was performed on the basis of Bray–Curtis distance by the R package “vegan” [30]. Meanwhile, non-parametric multivariate statistical analysis (Adonis, analysis of similarity [ANOSIM] and multi-response permutation procedure [MRPP]) was employed for comparing communities. Further, to evaluate the linkages between the prokaryotic community structure and environmental parameters, the Mantel test and redundancy analysis (RDA) were performed by the R package “microeco” [29]. LEfSe was employed for illustrating different taxa in sediment and water samples [31], calculated by the R package “microeco” [29]. 

Microbial ecological networks (MENs) were constructed by using the Molecular Ecological Network Analysis Pipeline (MENAP) (http://ieg4.ou.edu/MENA/ (accessed on 10 February 2022)) to reveal possible co-occurrence patterns [32,33]. Random matrix theory (RMT) threshold was set as 0.85 to construct MENs. For each node, within-module connectivity (*Z_i_*) and among-module connectivity (*P_i_*) [34] were calculated and used for the classification of its topological roles in the network. To identify the keystone taxa, the following simplified classification was established: (i) peripheral nodes (*Z_i_* ≤ 2.5, *P_i_* ≤ 0.62), which possessed only a few links that were almost always associated with nodes within their modules; (ii) connectors (*Z_i_* ≤ 2.5, *P_i_* > 0.62), which were highly connected to several modules; (iii) module hubs (*Z_i_* > 2.5, *P_i_* ≤ 0.62), which were highly connected to numerous microbes in their own modules; (iv) network hubs (*Z_i_* > 2.5, *P_i_* > 0.62), which acted as both module hubs and connectors. Module hubs, connectors, and network hubs were referred to as keystone nodes [35,36]. We generated 1000 corresponding random networks with the same network size and an average number of links for each network. The visualization of MENs was performed by the R package “ggraph” (https://github.com/thomasp85/ggraph (accessed on 10 February 2022)). 

To investigate the assembly mechanisms of different microorganism groups, the Infer Community Assembly Mechanisms by Phylogenetic-bin-based null model (iCAMP, https://github.com/DaliangNing/iCAMP1 (accessed on 10 February 2022)) was employed [9]. By using iCAMP, five assembly mechanisms of different microorganism groups were obtained, including homogeneous selection (HoS), heterogeneous selection (HeS), dispersal limitation (DL), homogenizing dispersal (HD), and drift (DR). Besides, the variation between sediment and water groups in the saltern in HoS and DL was investigated in this study. The statistical difference test of stochasticity estimated between sediment and water groups was calculated by using the Mann–Whitney U test. 

### 2.5. Nucleotide Sequence Accession Numbers

The 16S rRNA gene data sets of Wendeng salterns determined in this study was deposited in the Sequence Read Archive under accession numbers PRJNA559148 and PRJNA799174 for all samples. 

## 3. Results

### 3.1. General Features of 16S rRNA Gene Sequences and Taxonomic Compositions of the Prokaryotic Communities

**Figure 2:**
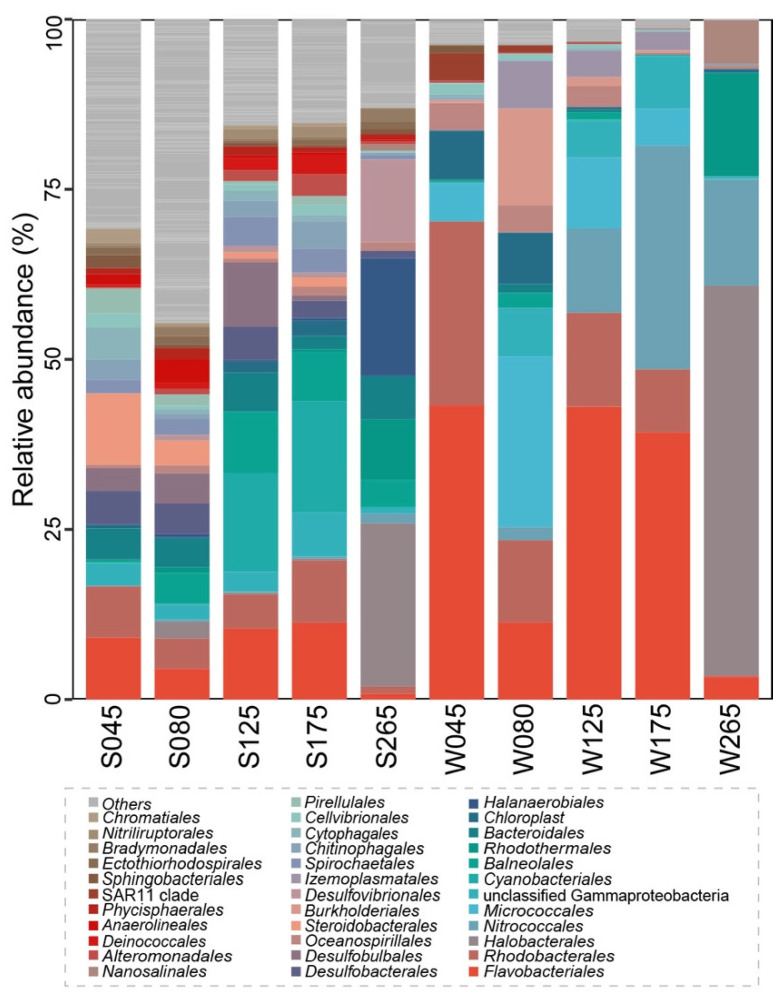
Prokaryotic community composition indicating the most abundant orders along a gradient of increasing salinity. The top 35 most abundant order taxa are shown. The labels S and W represent sediment and water samples, respectively. The number in each sample means salinity.

After sequences filtering, clean data were obtained for a total of 543,086 sequences (length distribution of valid sequences was 361–402 bp), generating 5221 OTUs. The 14 most abundant phyla represented 81.39–93.96% and 92.08–98.21% of the prokaryotic community composition in sediment and water samples obtained from Wendeng salterns (Figure 1, Table S1). The phyla *Bacteroidota* and *Proteobacteria* were the most abundant in the sediment and water samples from salterns. The phylum *Halobacterota* showed a hump dependence on salinity in the sediment samples; the relative abundance of phylum *Halobacterota* in the sediment was higher than in water samples in low salinity. The phyla *Chloroflexi* showed a negative dependence on salinity in both sediment and water samples. At the order level, the relative abundance of the top 35 most abundant order taxa exhibited different patterns in sediment and water samples (Figure 2). In detail, the orders *Halobacterales* and *Nitrococcales* were the most abundant taxa in sediment samples (salinity 265 g/L); by contrast, the orders *Omnitrophales*, *Cytophagales*, and *Halobacterales* were the most abundant taxa in water samples (salinity 265 g/L). The relative abundance of the order *Halobacterales* showed a hump-shaped dependence on salinity in both sediment and water samples, while, the relative abundance of the order *Erysipelotrichales* presented a similar pattern only in the water samples. Further, the variation of the order *Rhodobacterales* exhibited a more obvious hump-shaped dependence on salinity in sediment than in water samples. The orders *Flavobacteriales*, *Planctomycetales*, and *Spirochaetales* revealed a negative dependence on salinity in water samples. 

### 3.2. Diversity of Prokaryotic Communities and Relationships with Physicochemical Factors

**Figure 3:**
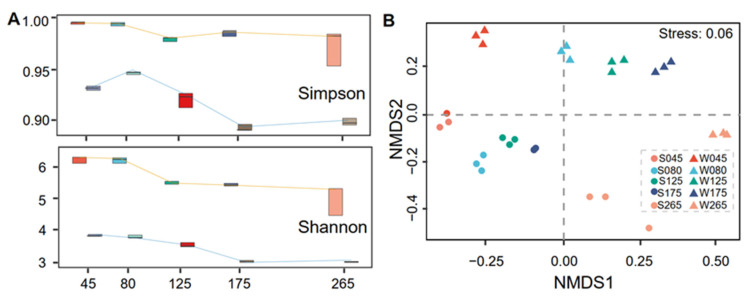
The biodiversity of the prokaryotic community. (**A**), The α diversities (Simpson and Shannon index) were analyzed by R package (microeco) and are displayed along a gradient of increasing salinity. The orange and blue lines represent sediment and water groups in salterns, respectively. (**B**), Non-metric multidimensional scaling (NMDS) was performed to exhibit the β-diversity of the prokaryotic community in salterns.

The α-diversities (Shannon, Simpson, and phylogenetic-diversity [PD] indexes) differed significantly between sediment and water samples obtained from Wendeng salterns along a gradient of increasing salinity (Figure 3A). Meanwhile, the α-diversities in the same salinity ponds also exhibited a significant difference between sediment and water samples (Table S1). On the basis of NMDS, the succession of prokaryotic communities along increasing salinity showed a very similar pattern in sediment and water samples (Figure 3B). The community structures of sediment and water samples were also easily distinguished (Table S2). The shared OTUs in the sediment samples were more than in the water samples (Figure S1A,B). By contrast, the number of shared OTUs in ponds with the same salinity exhibited a diminishing tendency along with increasing salinity (Figure S1C–G), and the rarefaction curve on basis of Shannon and Simpson indexes was obviously different (Figure S1H,I). The significantly differential abundant family taxa differed in the same salinity ponds (Figures S2 and S3). The families *Woeseiaceae*, *Cyclobacteriaceae*, *Pirellulaceae*, *Desulfocapsaceae*, *Ilumatobacteraceae* and *Bacteroidetes*_BD2−2 were abundant in sediment samples at salinity of 45 g/L; by contrast, the families *Rhodobacteraceae*, *Cryomorphaceae*, and Clade_I_o_SAR11 were abundant in water samples at salinity of 45 g/L. The families *Rhodobacteraceae*, *Saprospiraceae*, *Marinobacteraceae*, and *Trueperaceae* were abundant in sediment samples at salinity of 175 g/L; by contrast, only the family *Nitrococcaceae* was abundant in water samples at salinity of 175 g/L. In addition, the families *Haloferacaceae* and *Rhodothermaceae* were abundant in both sediment and water samples at salinity of 265 g/L. 

**Figure 4:**
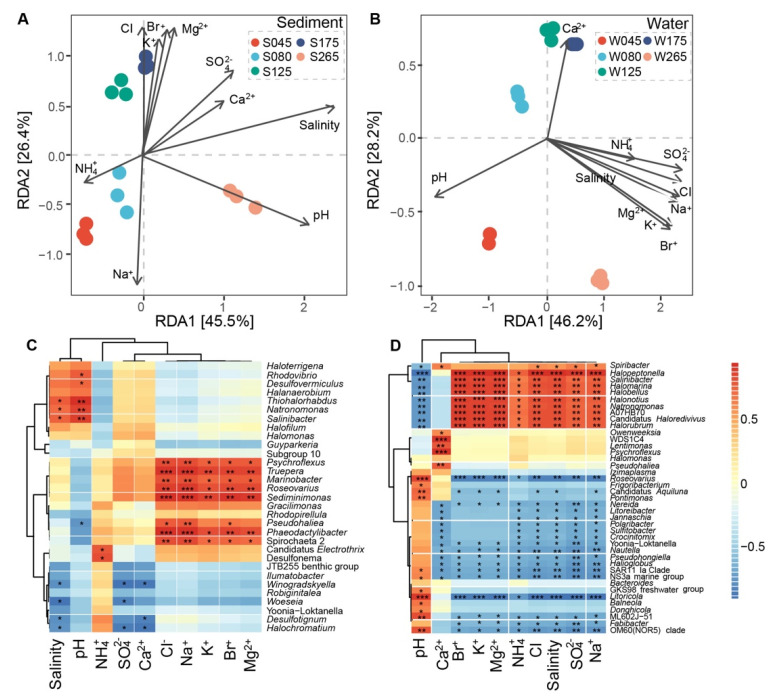
Relationships between physicochemical factors and prokaryotic community composition. (**A**,**B**), Redundancy analysis (RDA) between prokaryotic community bray-distance and physicochemical factors was employed for both sediment and water samples of the salterns. (**C**,**D**), The genera strongly related to physicochemical factors are shown; (**C**), sediment groups; (**D**), water groups. Significant differences are marked. (* 0.01 < *p* ≤ 0.05, ** 0.001 < *p* ≤ 0.01, *** *p* ≤ 0.001).

To further illustrate the variation of prokaryotic communities, we measured a series of physicochemical factors (Table S2). On the basis of RDA, salinity and pH were the most important factors that influenced the community structure at salinity of 265 g/L in the sediment samples (Figure 4A,B and Table S3). The community structure of sediment and water samples at the same salinity appeared influenced by different physicochemical factors. The genera *Natronomonas*, *Thiohalorhabdus*, and *Salinibacter* showed a significantly positive relationship with salinity and pH in the sediment samples; by contrast, the genera *Woeseia*, *Winogradskyella*, *Halochromatium*, and *Desulfotignum* showed a significantly negative relationship with salinity (Figure 4C). Interestingly, the genera *Spiribacter*, *Halopeptonella*, *Salinibacter*, *Halomarina*, *Halobellus*, *Halonotius*, *Natronomonas*, and *Halorubrum* exhibited a significantly positive relationship with salinity, but a negative association with pH in the water samples; by contrast, the genera *Roseovarius* and *Litoricola* showed a significantly negative relationship with salinity, but a positive relationship with pH (Figure 4D). α-diversity mostly showed a significantly negative relationship with salinity and pH in the sediment samples (Figure S4A), but a significantly positive relationship with pH in the water samples (Figure S4B). In addition, α-diversity mostly presents a significantly negative relationship with physicochemical factors, except pH (Figure S4B). 

### 3.3. Characters of MENs

**Figure 5:**
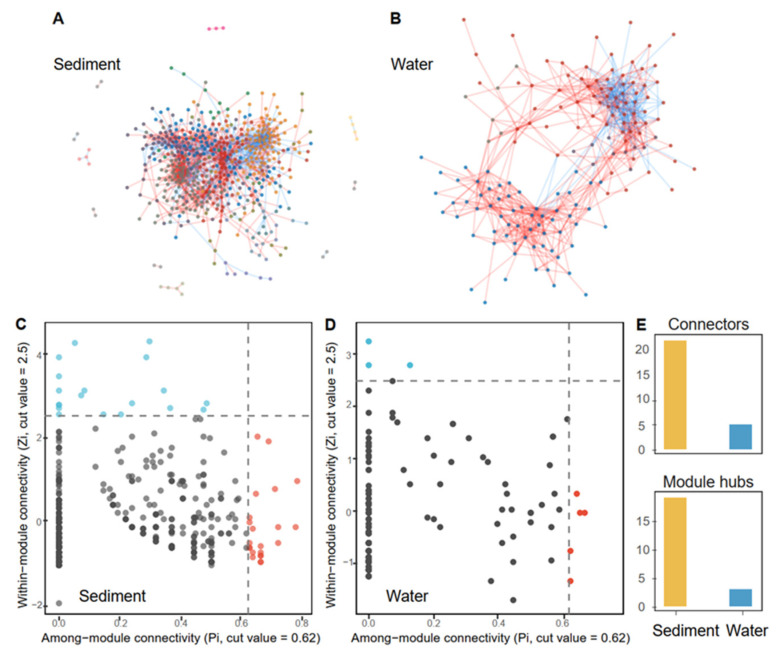
Salterns networks in sediment and water samples. (**A**,**B**), Visualization of the microbial MENs in sediment and water groups. Modules are randomly colored. The red and blue links between nodes represent positive and negative relationships, respectively. (**C**,**D**), The role of OTUs in network communities were determined by within-module connectivity (*Z_i_*) and among-module connectivity (*P_i_*). The light blue and red points represent module hubs and connectors, respectively. (**E**), Summary of keystone taxa (including module hubs and connectors).

We constructed MENs of the sediment and water samples (Figure 5A,B) on the basis of Pearson correlations of log-transformed operational taxonomic unit (OTU) abundances. The empirical MENs of both sediment and water samples were significantly different from random MENs, and all empirical MENs exhibited scale-free features (Table S4). Degree distributions of MENs both in sediment and in water samples followed the power-law distribution, indicating “rich get richer”. MENs of the sediment samples were more complex than those of the water samples. MENs of the water samples possessed higher average degree (avgK), average clustering coefficient (avgCC), average path distance (APD), graph density (GD), transitivity (Trans), and positive links along with diminishing networks composition. 

The putative roles of network nodes were confirmed on the basis of their within-module connectivity (*Z_i_*) and participation coefficient (*P_i_*) (Table S5). Most nodes were identified as peripheral (92.6%, 598/646), and the remaining nodes were module hubs and connectors. Due to the contribution of module hubs and connectors to network topology, module hubs and connectors have been proposed to represent potential keystone taxa. The keystone taxa of MENs in the sediment samples were more than those in the water samples (Figure 5C,D). The bulk of keystone taxa of MENs in the sediment samples was affiliated to *Rhodobacteraceae* (4), *Spirochaetaceae* (3), *Balneolaceae* (2), *Flavobacteriaceae* (2), and *Saprospiraceae* (2) (30.2%, 13/43). The order, to which keystone taxa in MENs of sediment were affiliated, was more diverse than in the water samples (Table S5; 27:5). The family *Woeseiaceae*, reported as the most abundant in marine sediment, could contribute to network topology as a module hub of MENs in the sediment samples. 

### 3.4. Dispersal Limitation and Homogeneous Selection Shape Prokaryotic Communities in Saltern Sediment

**Figure 6:**
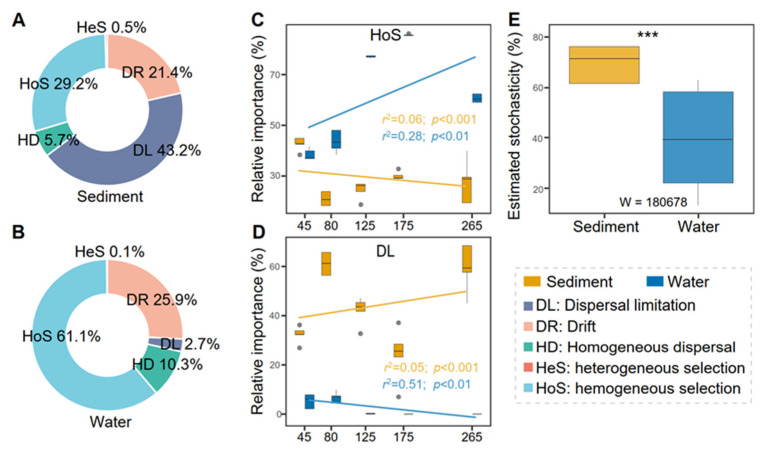
Relative importance of different ecological processes in salterns’ sediment and water. (**A**), Relative importance of different ecological processes in sediment samples. (**B**), Relative importance of different ecological processes in water samples. (**C**,**D**) Changes of homogeneous selection and dispersal limitation in sediment (orange box) and water (blue box). The adjusted *r*^2^ and *p* values from linear regressions are shown. (**E**) Stochasticity estimated in both sediment and water samples. Mann–Whitney U test results are shown, and significance is expressed as *** *p* ≤ 0.001.

We employed iCAMP to infer community assembly mechanisms and found that dispersal limitation (DL) and homogenous selection (HoS) were the key processes driving prokaryotic community assembly in saltern sediment, but HoS was the most essential process in saltern water (Figure 6A,B). DL had a more significant effect on community assembly in saltern sediment (33.20%), followed by HoS (39.65%); by contrast, HoS exerted the most prominent effect in saltern water (54.17%). The variation of HoS in saltern water exhibited a rising trend along with increasing salinity and a slightly increasing tendency in saltern sediment (Figure 6C). The changes of DL were characterized by a slightly diminishing trend along with salinity in saltern sediment but maintained a low contribution to community assembly in saltern water (Figure 6D). Estimated stochasticity between saltern sediment and water samples exhibited significant differences on the basis of the Mann–Whitney U test (Figure 6E).

## 4. Discussion

The effects of salinity on the structure of the prokaryotic community are mainly confined to solar saltern ponds, salt lakes, dynamic estuaries, and vertical water columns. Some studies have focused on multi-pond solar salterns with different salinity levels [12,37,38]. However, these reports did not focus on how salinity affects microbial community assembly processes. In this study, we determined the effects of environmental factors on the dynamics of the prokaryotic community composition in sediment and water samples from multi-pond solar salterns. Our results broaden the understanding of the precise microbial structure patterns in response to gradients of salinity and the microbial community assembly rules through a deep analysis involving different sources of samples (water and sediments), environmental parameters, and the quantification of the relative importance of ecological processes. 

The diversity of prokaryotic communities in a low-salinity saltern was higher than that in a high-salinity saltern, consistent with the common ecological principle according to which extreme environments are associated with a low community diversity [12,39]. The possible explanation for this negative effect could be attributed to the fact that the accumulation of salt in water and sediment environments elevates extracellular osmolarity [40], and microorganisms that fail to adapt to osmotic stress may die, thus reducing microbial α diversity. The variation in microbial community structure of a solar saltern was also mainly explained by salinity in this study, which is consistent with the results found for estuarine and marine environments [15]. In contrast, this diversity did not decrease as salinity increased up to low salinity values of <80 g/L (Table S1). Like in other studies, the prokaryotic community diversity did not decrease with the increase in salinity within a range of 0–100 g/L, as observed in salt lakes [4,41]. Furthermore, the prokaryotic community diversity in sediment samples was much higher than in water samples; similar results were reported for a Tunisian multi-pond solar saltern [37]. This may have occurred because the sediment contained a stable and nutrient-rich environment.

The presence of diverse communities in water and sediments was also shown by network analysis. Microbial network analysis can improve our perspectives on ecological processes and complex interaction webs beyond microbial community composition and richness [42]. Microbial co-occurrence network dynamics appeared more complex in the sediment than in the water of salterns. One potential reason is that communities in sediments had a higher Simpson and Shannon index than in water samples. A previous study focused on the co-occurrence networks in a mountain ecosystem also found that low bacterial diversity had low network complexity, which supports our results [43]. Another potential reason is that the sediment contains a stable environment, while water in salterns might be frequently affected by tides and sun exposure. Indeed, studies showed that eukaryotic plankton co-occurrence networks were influenced by distinct environmental factors in reservoirs [44]. In the network, keystone taxa have been frequently referred to as “ecosystem engineers” owing to their enormous influence in the community [32]. In the saltern system, sediment harbored much more keystone taxa, most of which belonged to *Woeseiaceae*, *Rhodobacteraceae*, and *Flavobacteriaceae*. Among these groups, *Woeseiaceae* has been identified as an abundant core member of microbial communities in global marine sediments [45,46], suggesting that *Woeseiaceae* might have a large adaptability to salinity. 

The community assembly mechanism is one of the most compelling questions in ecology, and previous studies have indicated that assembly mechanisms mainly include deterministic and stochastic processes [8]. The deterministic processes included homogeneous selection (HoS) and heterogeneous selection (HeS), while the stochastic processes comprise dispersal limitation (DL), homogenizing dispersal (HD), and drift (DR) [8]. It is commonly known that salinity imposes an intense selection pressure on the microbial community, which results in the dominance of deterministic processes in coastal wetland [1] and desert [3] ecosystems. However, our study found that differently from the saltern water system and other soil ecosystems [1,3], salinity could impose strong dispersal limitation processes on the microbial community of saltern sediment samples. One possible reason is that microorganisms in the sediment of different saltern pools have a poor dispersal ability than in water. Another reason is that sediment has a stable and nutrient-rich environment, and salt-tolerant microorganisms could respond rapidly to environmental changes and divide, thereby reducing the environmental heterogeneity gradient.

Microbial biodiversity studies have led to two major conflicting hypotheses [47]. One is the “seed bank” hypothesis, suggesting that microorganisms are ubiquitous and have few barriers to gene flow, which results in similar microbial communities across different spatial scales and habitats [21,48]. The other one is the ‘‘barriers to dispersal’’ hypothesis, which shows similar patterns in animals or plants, suggesting that microorganism’s differentiation is governed by geographic barriers or ecological barriers [22,49]. Many studies reported the relationship between microbial community diversity and different hypersaline environments [4,50], supporting the ‘‘barriers to dispersal’’ hypothesis. As one of the studies of microbial community diversity in different hypersaline backgrounds, our work reports the presence of members of *Halobacterota*, *Proteobacteria*, *Bacteroidota*, *Planctomycetota*, *Spirochaetota, Nanohaloarchaeota*, *Chloroflexi*, *Hydrogenedentes*, KSB3 (*Modulibacteria*), and *Latescibacteria* in sediment and water samples of solar salterns. Some common members of prokaryotic communities were also reported in other hypersaline environments, such as *Chloroflexi*, *Proteobacteria*, *Planctomycetota*, and *Spirochaetota* and *Halobacteriales* [51,52,53,54,55]. These results show that different hypersaline environments (e.g., solar salterns and salt lakes) might contain some common members of Bacteria or Archaea, which supports the “seed bank” hypothesis. Furthermore, in this study, all salterns originated from seawater and comprised a set of shallow ponds, where water evaporates, and salts concentrate. Thus, the initial composition of the prokaryotic community should have been similar in each saltern. However, as the water evaporated, and different salinities were achieved, different prokaryotic community compositions appeared in salterns depending on their salinity (Figure 3). These results also support the “seed bank” hypothesis, according to which “everything is everywhere, but the environment selects” [48]. However, the mechanism driving the “seed bank” process requires further study. Additionally, the main composition of the prokaryotic communities of Wendeng solar salterns differs from that of other salterns, such as a Greek solar saltern [56]. Therefore, geographic and ecological barriers may be the governing powers that create and maintain biodiversity in the Wendeng solar salterns, developing the unique microbial community structure in this unique eco-system.

In aquatic systems, such as salt lakes [4], solar saltern ponds [12], and the Baltic Sea [15], salinity is a major environmental driving force to control prokaryotic communities. Global studies have found that salinity, rather than other physical and chemical factors, determines microbial community composition [1,3,5,57,58,59]. Thus, microbial studies considering salinity gradients may provide more clues to the global distribution pattern of microbial communities depending on salinity changes. In this study, the Mantel test showed that both salinity and pH were the most critical environmental factors to regulate prokaryotic structure changes in the sediment and water of salterns. Consistent with our reports, many other reports have shown that pH is the main driving force of microbial community distribution in different ecosystems [60]. pH has been suggested to be the main factor that influences the physiochemical status of aquatic ecosystems [61]. 

In summary, our results showed that the composition of prokaryotic communities and assembly processes in Wendeng salterns were directly or indirectly determined by salinity. Our findings shed light on the distribution pattern of prokaryotic communities in relation to the salinity gradient of the whole community, in a single evolutionary process. This information will help to predict the ecological responses of future environmental changes and help to reveal the global microbial distribution in relation to salinity gradients.

## Data Availability

Not applicable.

## Supplementary Materials

The following are available online at https://www.mdpi.com/article/10.3390/genes13020385/s1, Figure S1: Venn diagrams showing the shared and unique OTUs, Figure S2: Differential abundance test for the sediment of salterns, Figure S3: Differential abundance test for the water of salterns, Figure S4: Relationships between physicochemical factors and α diversities of prokaryotic communities, Table S1: Relative abundance of phylum taxa in both sediment and water samples, Table S2: Characterization of physicochemical factors in sediment and water samples, Table S3: Mantel test for Bray distance and physicochemical factors, Table S4: Characterization of networks in sediment and water groups, Table S5: Summary of putative roles in networks.

## References

[1] Zhang G.L., Bai J.H., Tebbe C.C., Zhao Q.Q., Jia J., Wang W., Wang X., Yu L. (2021). Salinity controls soil microbial community structure and function in coastal estuarine wetlands. Environ. Microbiol..

[2] Banda J.F., Zhang Q., Ma L., Pei L., Du Z., Hao C., Dong H. (2021). Both pH and salinity shape the microbial communities of the lakes in Badain Jaran Desert, NW China. Sci. Total Environ..

[3] Zhang K., Shi Y., Cui X., Yue P., Li K., Liu X., Tripathi B.M., Chu H. (2019). Salinity Is a Key Determinant for Soil Microbial Communities in a Desert Ecosystem. mSystems.

[4] Zhong Z.P., Liu Y., Miao L.L., Wang F., Chu L.M., Wang J.L., Liu Z.P. (2016). Prokaryotic Community Structure Driven by Salinity and Ionic Concentrations in Plateau Lakes of the Tibetan Plateau. Appl. Environ. Microbiol..

[5] Lozupone C.A., Knight R. (2007). Global patterns in bacterial diversity. Proc. Natl. Acad. Sci. USA.

[6] Caruso T., Chan Y.K., Lacap D.C., Lau M.C.Y., Mckay C.P., Pointing S.B. (2011). Stochastic and deterministic processes interact in the assembly of desert microbial communities on a global scale. ISME J..

[7] Nemergut D.R., Schmidt S.K., Fukami T., O’Neill S.P., Bilinski T.M., Stanish L.F., Knelman J.E., Darcy J.L., Lynch R.C., Wickey P. (2013). Patterns and Processes of Microbial Community Assembly. Microbiol. Mol. Biol. Rev..

[8] Zhou J., Ning D. (2017). Stochastic Community Assembly: Does It Matter in Microbial Ecology?. Microbiol. Mol. Biol. Rev..

[9] Ning D., Yuan M., Wu L., Zhang Y., Guo X., Zhou X., Yang Y., Arkin A.P., Firestone M.K., Zhou J. (2020). A quantitative framework reveals ecological drivers of grassland microbial community assembly in response to warming. Nat. Commun..

[10] Stomeo F., Valverde A., Pointing S.B., Mckay C.P., Warren-Rhodes K.A., Tuffin M.I., Seely M., Cowan D.A. (2013). Hypolithic and soil microbial community assembly along an aridity gradient in the Namib Desert. Extremophiles.

[11] Tripathi B.M., Stegen J.C., Kim M., Dong K., Adams J.M., Lee Y.K. (2018). Soil pH mediates the balance between stochastic and deterministic assembly of bacteria. ISME J..

[12] Benlloch S., López-López A., Casamayor E.O., Øvreås L., Goddard V., Daae F.L., Smerdon G., Massana R., Joint I., Thingstad F. (2002). Prokaryotic genetic diversity throughout the salinity gradient of a coastal solar saltern. Environ. Microbiol..

[13] Joint I., Henriksen P., Garde K., Riemann B. (2002). Primary production, nutrient assimilation and microzooplankton grazing along a hypersaline gradient. FEMS Microbiol. Ecol..

[14] Oren A. (2002). Molecular ecology of extremely halophilic Archaea and Bacteria. FEMS Microbiol. Ecol..

[15] Herlemann D.P., Labrenz M., Jurgens K., Bertilsson S., Waniek J.J., Andersson A.F. (2011). Transitions in bacterial communities along the 2000 km salinity gradient of the Baltic Sea. ISME J..

[16] Webb C.O., Ackerly D.D., McPeek M.A., Donoghue M.J. (2002). Phylogenies and community ecology. Annu. Rev. Ecol. Syst..

[17] Gibbons S.M., Caporaso J.G., Pirrung M., Field D., Knight R., Gilbert J.A. (2013). Evidence for a persistent microbial seed bank throughout the global ocean. Proc. Natl. Acad. Sci. USA.

[18] Gilbert J.A., Steele J.A., Caporaso J.G., Steinbruck L., Reeder J., Temperton B., Huse S., McHardy A.C., Knight R., Joint I. (2012). Defining seasonal marine microbial community dynamics. ISME J..

[19] Gilbert J.A., Field D., Swift P., Newbold L., Oliver A., Smyth T., Somerfield P.J., Huse S., Joint I. (2009). The seasonal structure of microbial communities in the Western English Channel. Environ. Microbiol..

[20] Caporaso J.G., Paszkiewicz K., Field D., Knight R., Gilbert J.A. (2012). The Western English Channel contains a persistent microbial seed bank. ISME J..

[21] Lennon J.T., Jones S.E. (2011). Microbial seed banks: The ecological and evolutionary implications of dormancy. Nat. Rev. Microbiol..

[22] Whitaker R.J., Grogan D.W., Taylor J.W. (2003). Geographic barriers isolate endemic populations of hyperthermophilic archaea. Science.

[23] Follows M.J., Dutkiewicz S., Grant S., Chisholm S.W. (2007). Emergent biogeography of microbial communities in a model ocean. Science.

[24] Rognes T., Flouri T., Nichols B., Quince C., Mahé F. (2016). VSEARCH: A versatile open source tool for metagenomics. PeerJ.

[25] Quast C., Pruesse E., Yilmaz P., Gerken J., Schweer T., Yarza P., Peplies J., Glöckner F.O. (2013). The SILVA ribosomal RNA gene database project: Improved data processing and web-based tools. Nucleic Acids Res..

[26] Yilmaz P., Parfrey L.W., Yarza P., Gerken J., Pruesse E., Quast C., Schweer T., Peplies J., Ludwig W., Glöckner F.O. (2014). The SILVA and “All-species Living Tree Project (LTP)” taxonomic frameworks. Nucleic Acids Res..

[27] Katoh K., Standley D.M. (2013). MAFFT Multiple Sequence Alignment Software Version 7: Improvements in Performance and Usability. Mol. Biol. Evol..

[28] Price M.N., Dehal P.S., Arkin A.P. (2009). FastTree: Computing large minimum evolution trees with profiles instead of a distance matrix. Mol. Biol. Evol..

[29] Liu C., Cui Y., Li X., Yao M. (2021). Microeco: An R package for data mining in microbial community ecology. FEMS Microbiol. Ecol..

[30] Dixon P. (2003). VEGAN, a package of R functions for community ecology. J. Veg. Sci..

[31] Segata N., Izard J., Waldron L., Gevers D., Miropolsky L., Garrett W.S., Huttenhower C. (2011). Metagenomic biomarker discovery and explanation. Genome Biol..

[32] Deng Y., Jiang Y.-H., Yang Y., He Z., Luo F., Zhou J. (2012). Molecular ecological network analyses. BMC Bioinform..

[33] Zhou J., Deng Y., Luo F., He Z., Tu Q., Zhi X. (2010). Functional molecular ecological networks. mBio.

[34] Guimerà R., Nunes Amaral L.A. (2005). Functional cartography of complex metabolic networks. Nature.

[35] Banerjee S., Schlaeppi K., van der Heijden M.G.A. (2019). Reply to ‘Can we predict microbial keystones’?. Nat. Rev. Microbiol..

[36] Röttjers L., Faust K. (2019). Can we predict keystones?. Nat. Rev. Microbiol..

[37] Baati H., Guermazi S., Gharsallah N., Sghir A., Ammar E. (2010). Novel prokaryotic diversity in sediments of Tunisian multipond solar saltern. Res. Microbiol..

[38] Dillon J.G., Carlin M., Gutierrez A., Nguyen V., McLain N. (2013). Patterns of microbial diversity along a salinity gradient in the Guerrero Negro solar saltern, Baja CA Sur, Mexico. Front. Microbiol..

[39] Fawley M.W., Fawley K.P., Buchheim M.A. (2004). Molecular diversity among communities of freshwater microchlorophytes. Microb. Ecol..

[40] Oren A. (2011). Thermodynamic limits to microbial life at high salt concentrations. Environ. Microbiol..

[41] Wu Q.L., Zwart G., Schauer M., Kamst-van Agterveld M.P., Hahn M.W. (2006). Bacterioplankton community composition along a salinity gradient of sixteen high-mountain lakes located on the Tibetan Plateau, China. Appl. Environ. Microbiol..

[42] Chen W., Wen D. (2021). Archaeal and bacterial communities assembly and co-occurrence networks in subtropical mangrove sediments under Spartina alterniflora invasion. Environ. Microbiome.

[43] Li J., Li C., Kou Y., Yao M., He Z., Li X. (2020). Distinct mechanisms shape soil bacterial and fungal co-occurrence networks in a mountain ecosystem. FEMS Microbiol. Ecol..

[44] Liu L., Chen H., Liu M., Yang J.R., Xiao P., Wilkinson D.M., Yang J. (2019). Response of the eukaryotic plankton community to the cyanobacterial biomass cycle over 6 years in two subtropical reservoirs. ISME J..

[45] Du Z.J., Wang Z.J., Zhao J.X., Chen G.J. (2016). Woeseia oceani gen. nov., sp nov., a chemoheterotrophic member of the order Chromatiales, and proposal of Woeseiaceae fam. nov. Int. J. Syst. Evol. Microbiol..

[46] Mussmann M., Pjevac P., Kruger K., Dyksma S. (2017). Genomic repertoire of the Woeseiaceae/JTB255, cosmopolitan and abundant core members of microbial communities in marine sediments. ISME J..

[47] Martiny J.B., Bohannan B.J., Brown J.H., Colwell R.K., Fuhrman J.A., Green J.L., Horner-Devine M.C., Kane M., Krumins J.A., Kuske C.R. (2006). Microbial biogeography: Putting microorganisms on the map. Nat. Rev. Microbiol..

[48] Gonnella G., Bohnke S., Indenbirken D., Garbe-Schonberg D., Seifert R., Mertens C., Kurtz S., Perner M. (2016). Endemic hydrothermal vent species identified in the open ocean seed bank. Nat. Microbiol..

[49] Fierer N., Jackson R.B. (2006). The diversity and biogeography of soil bacterial communities. Proc. Natl. Acad. Sci. USA.

[50] Tazi L., Breakwell D.P., Harker A.R., Crandall K.A. (2014). Life in extreme environments: Microbial diversity in Great Salt Lake, Utah. Extremophiles.

[51] Ley R.E., Harris J.K., Wilcox J., Spear J.R., Miller S.R., Bebout B.M., Maresca J.A., Bryant D.A., Sogin M.L., Pace N.R. (2006). Unexpected diversity and complexity of the Guerrero Negro hypersaline microbial mat. Appl. Environ. Microbiol..

[52] Sorensen K.B., Canfield D.E., Teske A.P., Oren A. (2005). Community composition of a hypersaline endoevaporitic microbial mat. Appl. Environ. Microbiol..

[53] Mani K., Taib N., Hugoni M., Bronner G., Bragança J.M., Debroas D. (2020). Transient Dynamics of Archaea and Bacteria in Sediments and Brine Across a Salinity Gradient in a Solar Saltern of Goa, India. Front. Microbiol..

[54] Akpolat C., Fernández A.B., Caglayan P., Calli B., Birbir M., Ventosa A. (2021). Prokaryotic Communities in the Thalassohaline Tuz Lake, Deep Zone, and Kayacik, Kaldirim and Yavsan Salterns (Turkey) Assessed by 16S rRNA Amplicon Sequencing. Microorganisms.

[55] Gorrasi S., Franzetti A., Ambrosini R., Pittino F., Pasqualetti M., Fenice M. (2021). Spatio-Temporal Variation of the Bacterial Communities along a Salinity Gradient within a Thalassohaline Environment (Saline di Tarquinia Salterns, Italy). Molecules.

[56] Tsiamis G., Katsaveli K., Ntougias S., Kyrpides N., Andersen G., Piceno Y., Bourtzis K. (2008). Prokaryotic community profiles at different operational stages of a Greek solar saltern. Res. Microbiol..

[57] Shi X., Zhao X., Ren J., Dong J., Zhang H., Dong Q., Jiang C., Zhong C., Zhou Y., Yu H. (2021). Influence of Peanut, Sorghum, and Soil Salinity on Microbial Community Composition in Interspecific Interaction Zone. Front. Microbiol..

[58] Rath K.M., Fierer N., Murphy D.V., Rousk J. (2019). Linking bacterial community composition to soil salinity along environmental gradients. ISME J..

[59] Mu D.S., Wang S., Liang Q.Y., Du Z.Z., Tian R., Ouyang Y., Wang X.P., Zhou A., Gong Y., Chen G.J. (2020). Bradymonabacteria, a novel bacterial predator group with versatile survival strategies in saline environments. Microbiome.

[60] Liu J., Fu B., Yang H., Zhao M., He B., Zhang X.H. (2015). Phylogenetic shifts of bacterioplankton community composition along the Pearl Estuary: The potential impact of hypoxia and nutrients. Front. Microbiol..

[61] Blodau C. (2006). A review of acidity generation and consumption in acidic coal mine lakes and their watersheds. Sci. Total Environ..

