# Supplementary material for: Salinity Gradient Controls Microbial Community Structure and Assembly in Coastal Solar Salterns
Source: Genes (Basel). 2022 Feb 21;13(2):385. doi: 10.3390/genes13020385 (PMC8872224; doi:10.3390/genes13020385)
Supplement: Supplementary file 1 [file genes-13-00385-s001.zip › supplementary figures.pdf]

Supplementary figures

Salinity gradient controls microbial community structure and assembly in coastal solar salterns

Tianran Song[1], Qiyun Liang[2], Zhaozhong Du[2], Xiaoqun Wang[2], Guan-Jun Chen[2], Zong-Jun Du[2]*, Da-Shuai Mu[1,2]*

[1] SDU-ANU Joint Science College, Shandong University, Weihai 264209, PR China

[2] College of Marine Science, Shandong University, Weihai 264209, PR China

Running title: Microbial community assembly in the solar saltern

*Address correspondence to:

Zong-Jun Du, E-mail: duzongjun@sdu.edu.cn; Tel. (+86) 06315688303. Mailing address: College of Marine Science, Shandong University (Weihai), 180 Wenhuaxi Road, Weihai, Box 264209, PR China

Da-Shuai Mu, E-mail: dashuai.mu@sdu.edu.cn; Tel. (+86) 06315688303. Mailing address: College of Marine Science, Shandong University (Weihai), 180 Wenhuaxi Road, Weihai, Box 264209, PR China

Conflict of Interest

No conflict of interest exists in the submission of this manuscript, and the manuscript has been approved by all authors for publication.

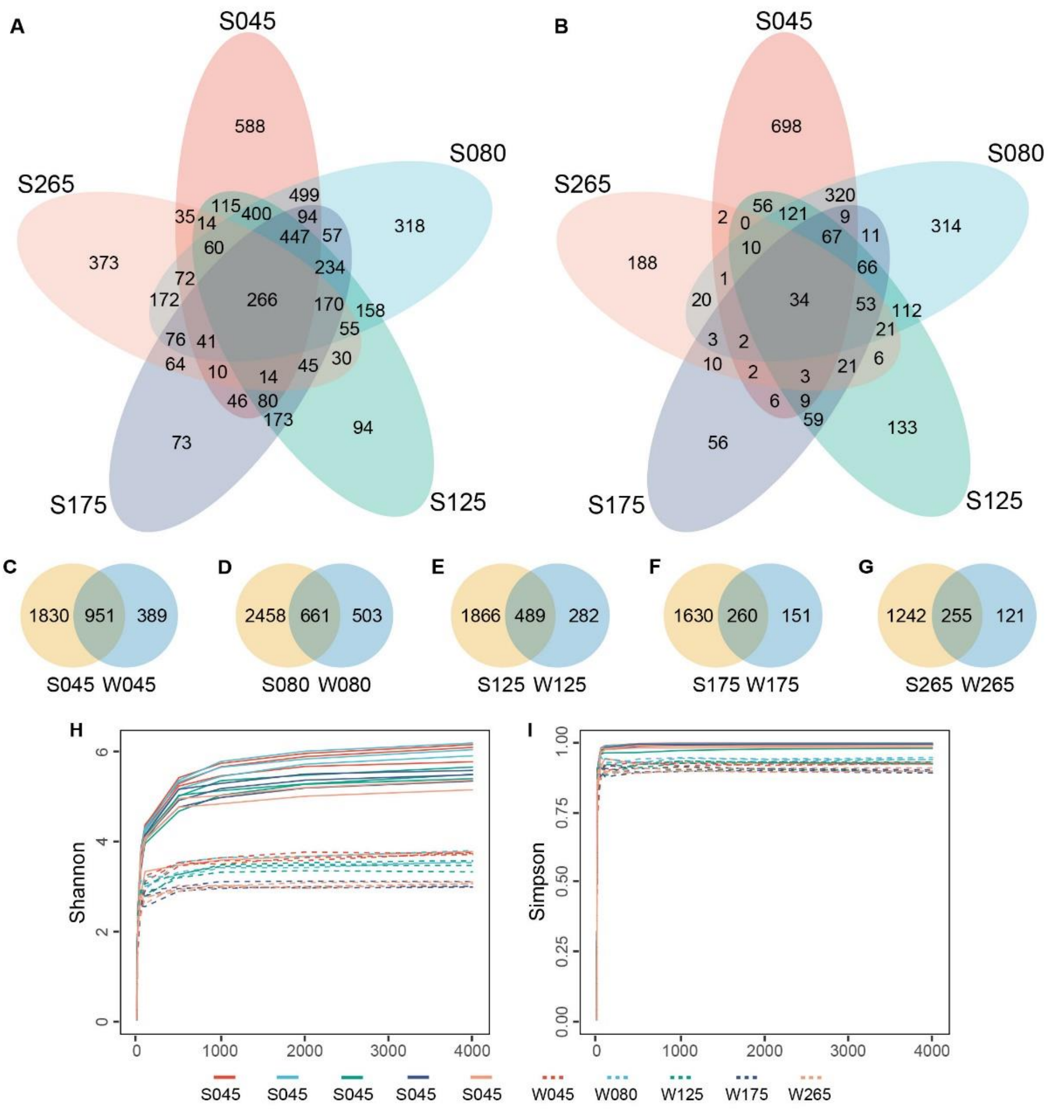

Figure S1. Venn diagrams showing the shared and unique OTUs.

A-B, Visualization of venn diagrams in sediment and water groups. **C-G**, Visualization of venn diagrams at same sampling site, comparing the shared and unique OTUs between sediment and water samples. **H-I**, Visualization of rarefaction curve on basis of Shannon and simpson index.

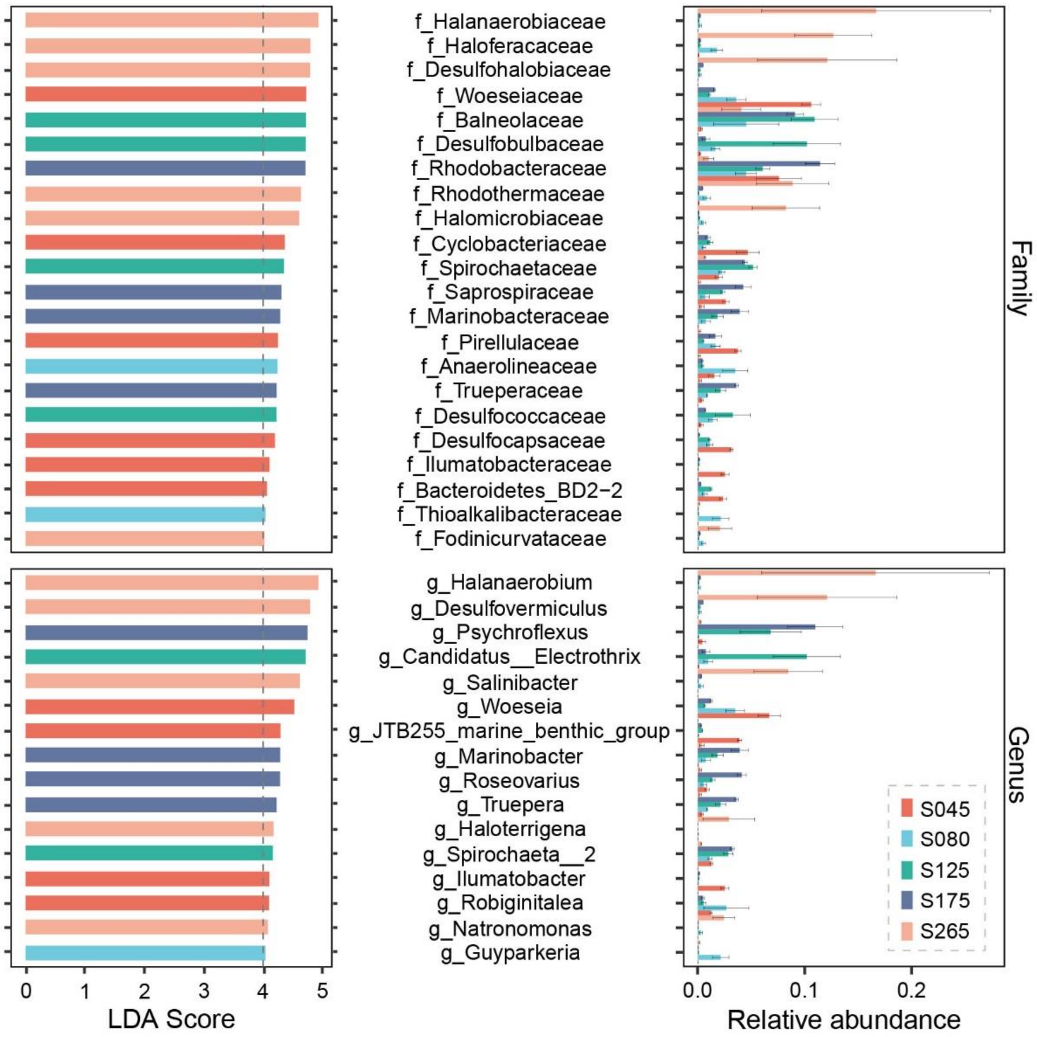

Figure S2. Differential abundance test in sediment of salterns.

The significant family and genus taxa, determining the community differences across groups, were confirmed by R package {microeco} on basis of LEFSe method.

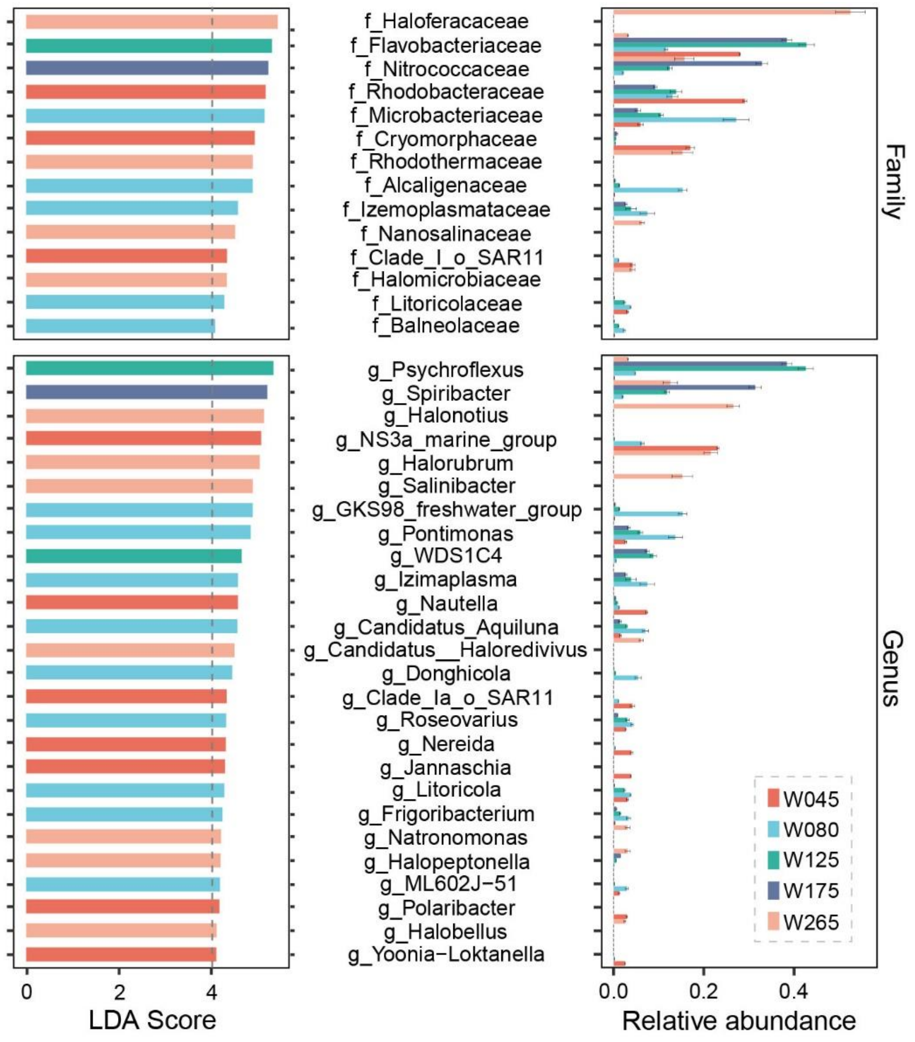

Figure S3. Differential abundance test in water of salterns.

The significant family and genus taxa, determining the community differences across groups, were confirmed by R package {microeco} on basis of LEFSe method.

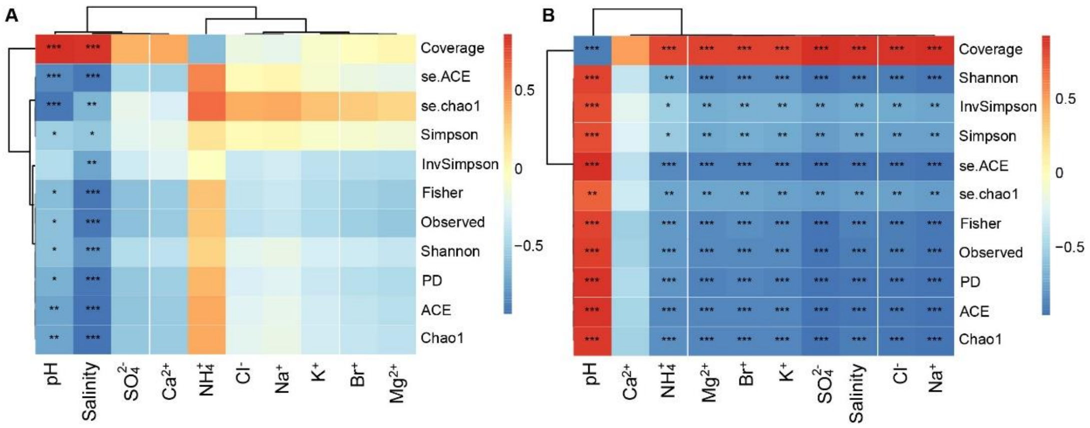

Figure S4. The relationships between physicochemical factors and alpha diversities of prokaryotic community.

A-**B**, The strong-related genus taxa with physicochemical factors were shown (A, sediment groups; B, water groups). Significant differences were marked. (***** $0.01 < P \leq 0.05$, ****** $0.001 < P \leq 0.01$, ******* $P \leq 0.001$)
